# Transoral Laser Microsurgery for Supraglottic Cancer

**DOI:** 10.3389/fonc.2018.00158

**Published:** 2018-05-09

**Authors:** Petra Ambrosch, Mireia Gonzalez-Donate, Asita Fazel, Claudia Schmalz, Jürgen Hedderich

**Affiliations:** ^1^Department of Otorhinolaryngology, Head and Neck Surgery, Christian-Albrechts-University Kiel, Kiel, Germany; ^2^Department of Radiation Oncology, Christian-Albrechts-University Kiel, Kiel, Germany; ^3^Institute of Medical Informatics and Statistics, Christian-Albrechts-University Kiel, Kiel, Germany

**Keywords:** supraglottic cancer, transoral laser microsurgery, larynx preservation, oncologic outcomes, functional outcomes

## Abstract

**Introduction:**

Transoral laser microsurgery (TLM) is an accepted and effective treatment strategy for supraglottic carcinomas. Early supraglottic carcinoma has excellent outcomes independently of the treatment approach. The role of TLM for the treatment of locally advanced tumors is debated. Particularly, the functional outcomes after TLM have to be proven by functional assessment of large cohorts of patients. This study analyzes the oncologic and functional outcomes after TLM for supraglottic carcinomas.

**Patients and methods:**

Ninety-one patients with pT1-pT4a supraglottic carcinomas treated between January 2002 and December 2012 were analyzed. Distribution of tumors (UICC 2010) was 11 patients with pT1, 31 patients with pT2, 36 patients with pT3, and 13 patients with pT4a tumors. Node status was positive in 40 (43.6%) patients; 61 (67.1%) patients had stage III or IVa disease. Local control and survival were estimated using the Kaplan–Meier method. For the assessment of functional outcomes, the MD Anderson Dysphagia Inventory (MDADI), the Voice Handicap Index-10 (VHI-10), and the performance status scale for head and neck cancer [Performance Status Scale for Head and Neck (PSS-HN)] were used.

**Results:**

The median age was 62 years (range, 33–88 years). Fourteen (15.4%) patients developed a local or locoregional recurrence. The 5-year local control rate and 5-year ultimate local control rate were 72 and 92%, respectively. The 5-year overall survival rate was 63%. Twelve (13.2%) patients needed temporary tracheostomy. Sixty-eight (74.0%) patients had a nasogastric feeding tube post-operatively. At 1-year post-operative follow-up, only three patients were PEG dependent. The median VHI-10 score was 35, the median MDADI composite score was 80, and the median score of the domain “normalcy of diet” in the PSS-HN was 91.

**Conclusion:**

The oncologic outcomes are comparable to the results of open surgery for early and advanced supraglottic carcinomas. Functional swallowing outcome is superior to open surgery and to concomitant chemoradiation. Patients treated with TLM perceive low levels of voice- and swallowing-related quality of life impairment.

## Introduction

Currently, different treatment modalities are available for supraglottic carcinomas. Oncologic outcome is still the most important treatment goal. However, the preservation of a functioning larynx and issues of quality of life (QoL) have become more important in recent years and influence decision-making. There is consensus that early supraglottic carcinomas can be effectively treated by open-neck supraglottic laryngectomy (SGL), transoral laser microsurgery (TLM), and radiotherapy (RT). The first long-term results of 240 patients with early laryngeal carcinomas – among them 30 patients with supraglottic lesions – treated with TLM between 1979 and 1985 were published by Steiner in 1993 (1). In a subsequent report, the validity of TLM for the treatment of early supraglottic carcinomas could be shown (2). In recent years, TLM has gained approval for organ preservation surgery in early supraglottic tumors as a less invasive surgical method with superior functional results compared to external partial laryngectomies. In a systematic review, Swanson et al. (3) found that TLM performed better than IMRT for the treatment of early supraglottic cancer. The use of TLM for surgical larynx preservation in locally advanced supraglottic carcinomas, however, is still discussed controversially. A limited number of cohort studies confirm the oncologic and functional benefits of TLM (2, 4–14). With accurate preoperative diagnostics, appropriate patient selection, surgical expertise, and use of advanced technology adjuvant RT, TLM was shown to offer similar local control, larynx preservation, and survival as open-neck partial resections and similar survival as total laryngectomies. A recent meta-analysis of key oncological outcomes following TLM or open-neck conservation surgery for advanced T3–T4 laryngeal cancer confirmed both techniques as valid surgical options for larynx preservation (15). However, chemoradiotherapy protocols for larynx preservation have been examined in several randomized clinical trials (16–18) and a meta-analysis (19), thus making chemoradiotherapy an evidence-based and therefore preferred treatment approach in many cancer centers worldwide. However, recent studies have suggested that late toxicities are likely to contribute to high numbers of tumor-unrelated deaths years after treatment (17, 19). Another worrisome observation is that US cancer registry studies have shown that the increasing use of non-surgical larynx preservation strategies is accompanied by decreasing survival for patients with laryngeal cancer (20, 21). That fact was confirmed recently by data from Europe (22). Both observations raise the interest in larynx preservation surgery. The aim of this study is to present the oncologic and functional treatment results of TLM combined with selective neck dissection and adjuvant (chemo-)radiotherapy for early and locally advanced supraglottic carcinomas. The results are compared with results obtained with open-neck partial laryngeal resection and non-surgical larynx preservation with concurrent chemoradiotherapy.

## Patients and Methods

### Patients

This study was approved by the ethics committee of the Medical Faculty of Kiel University (D 417/14). Between January 2002 and December 2012, a total of 170 Patients with previously untreated squamous cell cancer of the supraglottic larynx were treated at the Department of Otorhinolaryngology, Head and Neck Surgery of Kiel University, Germany. Seventy-nine patients were excluded from the analysis with the following exclusion criteria: 17 patients presented with a previous malignant tumor, 9 patients were diagnosed with a simultaneous second primary tumor, 3 patients were diagnosed with simultaneous distant metastases, and 2 patients were diagnosed with non-resectable N3 metastases in the neck. Fifteen patients underwent concurrent chemoradiotherapy and another 22 patients were treated with total laryngectomy due to advanced local disease (T3 and T4a) with no option for non-surgical or surgical larynx preservation. Ten patients with very advanced locoregional disease were treated in palliative intent, and one patient was lost for follow-up. Ninety-one consecutively recruited patients were treated with frontline TLM. None underwent open-neck SGL, and none had to be excluded for technical reasons, due to insufficient endoscopic accessibility of the tumor. Of those 91 patients, 69 (75.8%) patients were male and 22 (24.2%) patients were female. The median age was 62 years (range, 33–88 years), and 17 (18.7%) patients were older than 70 years. Seventy-nine (86.8%) patients were active or former smokers with a median tobacco consumption of 35 pack years, 61 (67.0%) patients consumed alcohol regularly, and 12 (13.2%) patients were ex-alcoholics. Patient characteristics are shown in Table 1.

**Table 1:** Patient characteristics.

Characteristic	*N*	%
**Sex**		
Male	69	75.8
Female	22	24.2

**pT category**		
pT1	11	12.0
pT2	31	34.0
pT3	36	39.5
pT4a	13	14.5

**pN category**		
N0/pN0	51	56.0
pN1	9	10.0
pN2a	1	1.0
pN2b	15	16.5
pN2c	15	16.5
pN3	0	0

**UICC stage**		
I	8	8.8
II	22	24.2
III	26	28.6
IVa	35	38.5

**Smoking status**		
Never	12	13.2
Ever	79	86.8

**Alcohol consumption**		
Never	19	20.8
Regular	61	67.0
Ex-alcoholic	12	13.2

**Surgical margins**		
Negative (R0)	82	90.1
Microscopic positive (R1)	1	1.1
Uncertain	8	8.8
Macroscopic positive (R2)	0	0

### Preoperative Examination

Routine preoperative examination consisted in flexible laryngoscopy with adequate documentation. Accessibility of the tumor and tumor extent were determined with a staging microlaryngoscopy. All patients had panendoscopy for the exclusion of second primary tumors in the upper aerodigestive tract and a biopsy for tissue diagnosis. CT or MRI scans were routinely performed in all patients, except in T1 primary lesions. For staging of the neck, ultrasound and fine needle aspiration cytology were used, additionally to CT or MRI scans. Distant metastases were excluded by chest X-ray and ultrasonography of the abdominal organs. Pulmonary function tests were not routinely used.

### Operative Technique

The operation was performed in the technique previously described (2, 23). Briefly, small lesions of the suprahyoid epiglottis or ventricular fold were excised en bloc. Carcinomas involving the infrahyoid epiglottis and the ventricular folds were exposed with a bivalved laryngoscope and removed piecemeal. In those cases, the preepiglottic fat was resected completely. Tumors with extension to the paraglottic space were removed with the resection of parts of the vocalis muscle. Tissues of the base of the tongue, piriform sinus, or one arytenoid cartilage were included in the resection if indicated. To prevent post-operative hemorrhage from the superior laryngeal arteries, the arteries were double-clipped. In most cases, tracheostomy was not needed because of little post-operative edema. Tracheostomy was considered in elderly patients with low pulmonary reserve, in patients with a bleeding diathesis (e.g., anti-coagulant medication and hemodialysis), or if bleeding from larger arteries was observed during surgery.

### Treatment of the Primary Tumor

All patients received TLM with the intent to completely remove the supraglottic carcinoma with microscopically negative margins (R0 resection). The resection was guided by the growth of the tumor. Resections were not classified according to the ELS classification system for supraglottic resections (24). The reasons are that despite the complexity of the classification system, not all resections are classifiable and that the system is not yet widely accepted. In 25 (27.5%) patients, a re-resection was performed because of positive (R1) or uncertain (Rx) resection margins. Only four of the 25 re-resection specimens contained carcinoma cells. Including re-resection, 78 (85.7%) of the 91 supraglottic carcinomas were resected with clear margins. Thirteen (14.3%) patients had microscopically positive (*n* = 5) or uncertain margins (*n* = 7). Ten of those had adjuvant (chemo-)radiotherapy, and three patients were clinically controlled. Five (5.5%) patients were prophylactically tracheotomized after extended supraglottic resection to secure the airway and to reduce the risk of developing pneumonia. All patients were decannulated. Pathologic staging according to the UICC 2010 classification was used for the primary tumor and the neck. The distribution of the histopathologic grading of the primary tumors was as follows: G1, 7.0%; G2, 68%; G3, 25%.

### Treatment of the Neck

Seventy-six (83.5%) patients had either unilateral (34; 37.4%) or synchronous bilateral (42; 46.2%) neck dissection. In clinically node-negative necks, selective neck dissection with removal of the lymph nodes of levels II and III and in clinically node-positive necks of levels II–IV was performed. Neck node metastases were histopathologically confirmed in 40 (44.0%) patients. Metastases with extranodal spread were detected in 15 of the 40 (37.5%) patients with node-positive necks.

### Adjuvant Treatment

Adjuvant RT was not indicated in patients with R0 resected primary tumors and with pN0 necks. Adjuvant RT was indicated in patients with microscopically positive resection margins (R1 resection), in patients with more than one lymph node metastases in the neck and/or metastases with extranodal spread. Radiation technique and chemotherapy changed over time. In the vast majority of patients, a conventional radiation therapy technique was used (“Fletcher-technique” with three-dimensional planning, 6-MeV photons of a linear accelerator). In all cases, post-operative RT was directed to the primary site and both sides of the neck.

Post-operative RT was performed in 30 (32.9%) patients, 18 (60.0%) of whom also received concurrent platinum-based chemotherapy. Adjuvant (chemo-)radiotherapy was performed in 3/30 (10%) patients in stages I and II and in 27/61 (44.2%) patients in stages III and IVa because of positive resection margins, more than one lymph node metastases in the neck or lymph node metastases with extranodal spread (Table 2). Extranodal spread of lymph node metastases and positive resection margins were regarded indications for adjuvant chemoradiotherapy. Chemoradiotherapy received 18 out of 19 patients with positive margins and/or ENS. Multiple neck node metastases and uncertain margins were not regarded indications for chemoradiotherapy. Treatment consisted in 60 Gy to the primary site after R0 resection and in 64 Gy after R1 resection and in patients with uncertain resection margins. The radiation dose to the node-negative neck (N0/pN0) was 50 Gy, to the node-positive neck (pN^+^) 54 Gy. Radiation dose was conventionally fractionated (2 Gy once daily, total treatment time, 6 weeks). Chemotherapy was carboplatin (70 mg/m^2^/week) in 14 patients or cisplatin (25 mg/m^2^, d1–d4, weeks 1, 3, and 6) in 4 patients.

**Table 2:** Treatment approach.

UICC stage	Surgical treatment		Combined treatment		Total
	*N*	%	*N*	%	
I	8	8.8	0		8
II	19	20.9	3	3.3	22
III	19	20.9	7	7.7	26
IVa	15	16.4	20	22.0	35
	61	67.0	30	33.0	91

### Assessment of Swallowing- and Voice-Related QoL

The MD Anderson Dysphagia Inventory (MDADI) was used for the assessment of swallowing-related QoL. The MDADI is validated for German language and is a self-administered questionnaire with 20 questions for the evaluation of the patient’s perception of swallowing-related QoL. Besides a global assessment (a single question), it comprises the following three subscales: physical subscale (six items), functional subscale (five items), and emotional subscale (eight items). The instrument was scored according the index paper (25). The minimum composite score is 20 (extremely low functioning), and the maximum composite score is 100 (high functioning). Thus, a higher MDADI composite score represents better function and better QoL. The Voice Handicap Index (VHI) was used for the assessment of voice-related QoL. The VHI questionnaire is also validated for German language and comprises 30 items in three subscales (physical, functional, and emotional). The instrument was scored according the index paper (26). Using a 5-point scale, the maximum score is 120 (extremely low functioning). Thus, a low VHI score represents better function and better QoL. The Performance Status Scale for Head and Neck (PSS-HN) was used for the assessment of subjective swallowing function (27). The PSS-HN is an examiner-rated instrument. The subscales “normalcy of diet,” “understandability of speech,” and “eating in public” are rated from 0 to 100, with higher scores indicating better performance. Functional morbidities with score ≤50 were considered as significant (28).

In April 2014, a cross-sectional study on patient-reported outcomes was performed. Forty-six patients were still alive. Five patients were excluded, three patients because of previous laryngectomy due to recurrence (*n* = 2) or functional failure (*n* = 1) and two patients because of permanent PEG feeding. Those five patients were regarded as functional failures with respect to the SGL, initially performed. Forty-one patients received the MDADI and VHI questionnaires. The questionnaires were handed out to the patients in the outpatient clinic, or mailed with a personalized cover letter. Patients were asked to complete and return the questionnaires. The questionnaires were completed by 28 (68.3%) patients (VHI) and 29 (70.7%) patients (MDADI). For functional assessment with the PSS-HN, 36 (87.8%) patients were interviewed by the treating clinician in the outpatient clinics.

### Statistical Methods

Data were recorded prospectively and kept in a database. Follow-up data were available of all patients. The median follow-up interval was 47.4 months (range, 2.5–124.7 months). Local control and survival were calculated according the Kaplan–Meier method. All data of patient-reported outcomes (MDADI, VHI, and PSS-HN scores) were expressed descriptively, compared between subgroups and displayed with box–whisker plots. Statistical tests were not used because of limited numbers in subgroups.

## Results

### Local Control

Fourteen (15.4%) patients developed local (eight patients) or locoregional (six patients) recurrences. Nine patients were successfully salvaged: seven patients with further TLM (2 with TLM + ND, one patient with TLM + RT), and two patients with total laryngectomy; five patients are alive and free of tumor; and two patients died from tumor-unrelated causes. In three patients, salvage therapy (two patients with total laryngectomy and one patient with definitive RT) was not successful and two patients declined further surgery or RT and were treated with palliative intent. The analysis of local and locoregional recurrences is shown in Table 3. The 5-year Kaplan–Meier local control rate was 72% [62%; 83%] for the entire cohort, 76% for pT1 and pT2, 68% for pT3, and 67% for pT3 and pT4a diseases. The 5-year definitive local control rate (including salvage therapy) was 92.3% [87%; 100%] for the entire cohort. The Kaplan–Meier estimates of local control and the corresponding 95% confidence intervals are shown in Table 4.

**Table 3:** Analysis of local and locoregional recurrences.

TNM	Time since surgery (months)	rTrN	Salvage treatment	Outcome
pT1pN0	16	rT1	TLM	Alive, NED
pT1pN0	87	rT1	TLM	Alive, NED
pT1pN0	63	rT1	TLM	Alive, NED
pT2 pN0	15	rT2	TLM	Alive, NED
pT2 pN0	11	rT4	TL	Alive, NED
pT3pN0	56	rT2	TLM + RT	Dead, tumor unrelated
pT3pN0	23	rT4	TL + RT	Alive, NED
pT3pN1	8	rT4	TL	Dead, tumor related
pT2pN0	20	rT1rN1	TLM + ND	Dead, tumor unrelated
pT3pN2a	10	rT4rN2c	RT	Dead, tumor related
pT3pN0	23	rT1rN1	TLM + ND	Alive, NED
pT3pN0	10	rT4rN2c	Declined	Dead, tumor related
pT4apN0	24	rT4rN2c	TL + RT	Dead, tumor related
pT4apN2c	6	rT2rN2c	Declined	Dead, tumor related

**Table 4:** Kaplan–Meier estimates of local control.

	Years	Local control rate (%)	95% confidence interval
pT1 and pT2	2	83	72; 96
	3	83	72; 96
	5	76	63; 92
pT3	2	72	58; 90
	3	72	58; 90
	5	68	54; 87
pT3 and pT4a	2	75	63; 89
	3	75	63; 89
	5	67	56; 85

### Larynx Preservation

In seven (7.7%) patients, larynx preservation failed because of oncologic, and in three (3.3%) patients, larynx preservation failed because of functional reasons. In 81/91 (89%) patients, a functioning larynx could be preserved. The larynx could be preserved in 10/11 (91%) patients with pT1, in 30/31 (97%) patients with pT2, in 32/36 (89%) patients with pT3, and in 9/13 (69%) patients with pT4a primary tumors.

### Regional Control

The overall recurrence rate in the neck was 5.5% (5/91 patients). Three (3.3%) patients developed late metastases in the pN0 neck: two patients were successfully salvaged and one patient died tumor related. Two (2.2%) patients developed recurrent neck metastases: one patient was successfully salvaged and one patient declined further treatment and died from neck recurrence.

### Distant Metastases

Six (6.6%) patients developed distant metastases (five patients in the lung and one patient in lung and liver) without local or neck recurrence after median 10 months (range, 1–17 months) after surgery. All patients, who developed distant metastases, had advanced neck disease at presentation (five patients pN2c and one patient pN2b). In none of the patients with local or locoregional recurrences, distant metastases have been diagnosed.

### Second Primary Tumors

Second primary tumors were diagnosed in 17 (18.7%) patients. The second primary tumor occurred in the head and neck region in five (vocal cord contralateral to supraglottic carcinoma, one; tonsil, three; outer ear canal, one) and in other organs in 12 (lung, seven; esophagus, one; thyroid gland, one; colon, two; urinary bladder, one) cases.

### Survival

At the end of follow-up, 46 patients were alive and tumor-free. Fourteen (15.4%) patients had died from cancer and 31 (34.0%) patients had died from tumor-unrelated causes, eight (8.8%) of whom due to second primary tumors. The 5-year Kaplan–Meier overall survival rate was 69% for stages I and II and 58% for stages III and IVa diseases. The 5-year Kaplan–Meier disease-free survival rate was 79% for stages I and II and 64% for stages III and IV diseases. The Kaplan–Meier estimates for overall and disease-free survival and the corresponding 95% confidence intervals are shown in Table 5 for stages I and II and in Table 6 for stages III and IVa diseases.

**Table 5:** Kaplan–Meier estimates of survival for stages I and II.

	Years	Survival rate (%)	95% confidence interval
Overall survival	2	90	80; 100
	3	78	64; 96
	5	69	53; 90
Disease-free survival	2	85	72; 100
	3	85	72; 100
	5	79	65; 98

**Table 6:** Kaplan–Meier estimates of survival for stages III and IVa.

	Years	Survival rate (%)	95% confidence interval
Overall survival	2	75	65; 87
	3	68	57; 81
	5	58	47; 73
Disease-free survival	2	71	59; 84
	3	69	57; 82
	5	64	52; 78

### Post-operative Complications

Two (2.2%) patients experienced post-operative endolaryngeal hemorrhage. In both cases, the bleeding vessel was coagulated endoscopically and one of whom was tracheotomized. Four (4.4%) patients developed aspiration pneumonia, two of whom were tracheotomized. Another four (4.4%) patients suffered from airway-compromising laryngeal edema and were tracheotomized to secure the airway. Altogether, a total of 12 (13.2%) patients had temporary tracheostomies. In five patients tracheostomy was performed electively and in seven patients due to various complications, listed earlier. There were no therapy-related deaths and no permanent tracheostomies.

### Functional Results

#### Swallowing Rehabilitation

Twenty-three (26%) patients needed no feeding tube, among them 18 of the 42 (43%) patients with pT1 and pT2 and five of the 49 (10%) patients with pT3 and pT4a primary tumors. The remaining 68 (74%) patients received a feeding tube at the end of surgery. The feeding tube was removed when no aspiration was endoscopically visible, median after 14 days. Two (2.2%) patients remained dependent on PEG feeding and one (1.1%) patient had a total laryngectomy for functional reasons.

#### Dysphagia-Related QoL

**Figure 1:**
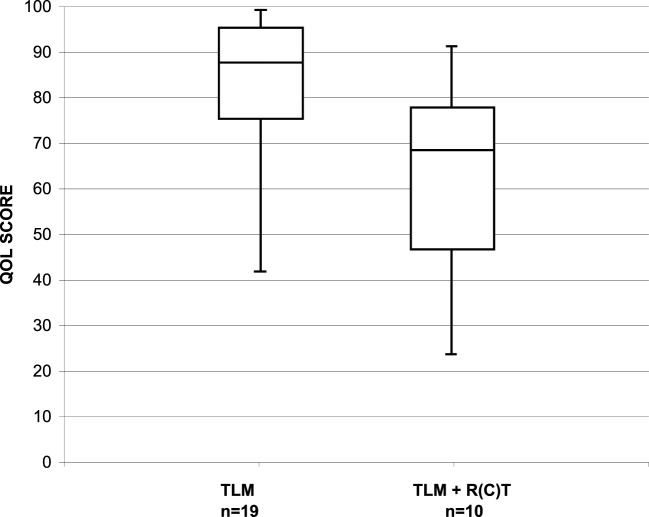
Distribution of the MDADI composite score in long-term survivors after TLM (*n* = 19) and after TLM + adjuvant RT (*n* = 10).

To evaluate patients’ account of their long-term swallow function, 41 patients alive and free of recurrence received the MDADI questionnaire. The questionnaire was fully completed by 29 patients. The median interval between completion of therapy and assessment was 88.5 months (range, 25–138 months; median: 75.7 months). The median MDADI composite score in the whole patient group was 80 (range, 30–100). Subgroup analysis showed that the parameters size of the primary tumor (pT1/pT2 vs. pT3/pT4a) had no relevant influence on the MDADI composite score, with 90 vs. 80. Relevant influence, however, had post-operative RT with a median MDADI composite score of 70 in irradiated and 90 in non-irradiated patients (Figure 1).

The PSS-HN instrument was completed for 36 patients. The median score for normalcy of diet was 91, that for eating in public was 85, and that for the understandability of speech was 90. The prevalence of functional deficits (score ≤50) was 8, 11, and 5%.

**Figure 2:**
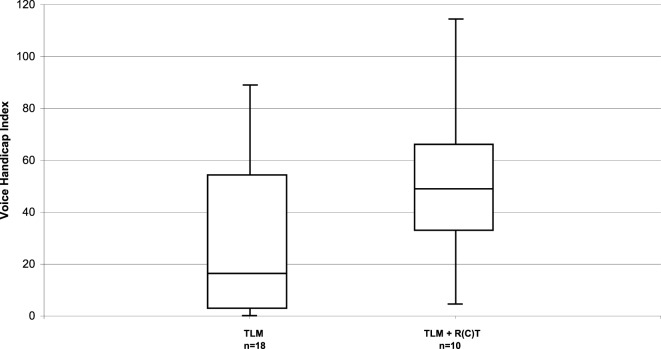
Distribution of the VHI in long-term survivors after TLM (*n* = 18) and after TLM + adjuvant RT (*n* = 10).

The VHI questionnaire was completed by 28 patients. The total VHI score in the whole patient group was 35 (range, 0–114). Few patients perceived high levels of voice handicap. Subgroup analysis showed that the parameters size of the primary tumor (pT1/pT2 vs. pT3/pT4a) had no relevant influence, with a median score of 23 vs. 37. Relevant influence, however, had post-operative RT with a median VHI score of 49 in irradiated and 16 in non-irradiated patients (Figure 2).

## Discussion

Since a national clinical practice guideline on diagnostic and treatment of laryngeal carcinoma was not available at the time, patients were treated, the treatment decisions were made in concordance with institutional guideline recommendations accepted at that time. From today’s knowledge, the potential value of adjuvant chemoradiotherapy in head and neck cancer patients with an elevated risk of recurrence is acknowledged and the modalities of adjuvant treatments have changed (29–31) also in our clinical practice.

It is generally agreed, however, that for the treatment of early supraglottic carcinomas (T1N0 and T2N0) single-modality therapy with the goal of curing the disease and preserving laryngeal functions is recommended. Early supraglottic carcinomas can be treated with RT or partial laryngeal resection with similar survival results (18). The surgical treatment options include SGL, either open-neck or TLM for the treatment of the primary tumor, together with uni- or bilateral, selective neck dissection. The non-surgical treatment is RT, preferably as IMRT, as single modality. Multimodality treatment with surgery and RT should be avoided because of added toxicity, poorer functional results, and increased necessity of total laryngectomy for salvage in the event of recurrence. In clinical practice, however, adjuvant RT in early stages is not always avoidable. In our cohort, three of the 30 (10%) patients with early supraglottic carcinomas had combined treatment because of positive or uncertain resection margins with no option for re-resection.

In selected cases of locally advanced supraglottic carcinomas (T3/T4a N0/N+), surgical larynx preservation, either by TLM or by open-neck SGL is an option. Contraindications for open-neck and endoscopic SGL in T3/T4a supraglottic carcinomas are bilateral paraglottic space invasion, bilateral vocal cord fixation, invasion of the thyroid cartilage at the glottic level, and extensive infiltration of the base of tongue, piriform sinus or soft tissues of the neck. With limited disease in the neck, the surgical treatment of the regional lymphatics consists in bilateral selective neck dissection. Depending on the histopathologic findings, regarding resection margins and status of the neck nodes, adjuvant radio- or chemoradiotherapy of the larynx and the neck might be indicated. In our patient cohort, 27 of the 61 (44.3%) patients with locally advanced supraglottic carcinomas needed combined treatment. The prevalence of adjuvant radio- and chemoradiotherapy varies among published TLM series between 22 and 41% and is mostly reported for the total patient cohort treated (8–10, 12, 14, 32).

### Oncologic Outcomes

There is evidence from cohort studies that the oncologic outcomes following TLM for early supraglottic carcinoma are comparable to open-neck SGL and to RT. The 5-year local control rate was 76% for pT1/pT2 tumors in our cohort. A high larynx preservation rate of 91% for pT1 and 97% for pT2 carcinomas could be achieved by early detection of local recurrences, which could be treated in five of the six cases by further larynx preserving TLM. The 5-year overall and disease-free survival rates for stages I and II were 69 and 79%, respectively. These results are comparable to other TLM series.

In a previous report, Ambrosch et al. (2) achieved a 5-year local control rate of 100% in pT1 and 89% in pT2 supraglottic tumors. Iro et al. (4) found 5-year local control rates of 95% for stage I and 88% for stage II disease. Grant et al. (5) reported on 38 patients with mainly early supraglottic carcinomas. The 2-year local control rate was 97 and 79% patients retained the larynx. Agrawal et al. (6) published a prospective phase 2 study in which 34 patients with T1/T2 supraglottic carcinomas were treated with TLM and post-operative RT. Only one patient was laryngectomized due to recurrence, but 9% failed because of functional reasons. Canis et al. (10) treated 277 patients with supraglottic carcinomas of all stages with TLM, among then 118 patients with pT1/pT2 lesions. The 5-year local control rate was 85%. Overall and recurrence-fee survival rates for stages I and II were 76 and 81%. Peretti et al. (8) achieved in 58 patients with pTis-pT2 disease a 5-year larynx preservation rate of 100%.

Two studies compare retrospectively open-neck SGL with TLM. In the series of Bussu et al. (7), larynx preservation and survival showed no significant difference in between treatment options, whereas functional results were better after TLM. Karatzanis et al. (33) compared oncologic and functional results of patients with early supraglottic carcinomas after TLM, open-neck SGL, or total laryngectomy. Local control and disease-specific survival were not significantly different. Functional outcomes were slightly better in the TLM group.

In our cohort, the 5-year local control rate was 68% for pT3 and 67% for pT3 and pT4a diseases. A functioning larynx could be preserved in 89% of patients with pT3 and in 69% patients with pT4a primary tumors. In the event of local recurrence, the auspices for salvage are compromised. Salvage treatment was successful in only two of the eight local/locoregional recurrences.

There are fewer reports on TLM for locally advanced supraglottic carcinomas in the literature. In the series of Iro et al. (4), local recurrence was diagnosed in 33% patients with T3 and in 10% patients with T4 carcinomas. Ambrosch et al. (34) treated 50 patients with pT3 supraglottic carcinomas with TLM. The 5-year recurrence-free survival and larynx preservation rates were 71 and 96%, respectively. Peretti et al. (8) reported on 20 patients with pT3 supraglottic carcinomas. The 5-year local control rate was 83%. In Vilaseca et al.’s (13) series of patients with T3 supraglottic carcinomas, the 5-year local control, overall, and disease-specific survival rates were 70, 46, and 62%, respectively. Steiner’s group reported in a retrospective review, long-term results of 104 pT3 and 55 pT4a patients with supraglottic carcinomas treated with TLM. Local or locoregional recurrences were observed in 20% of pT3 and in 22% of pT4a cases. The 5-year larynx preservation rate was 82% for pT3 and 76% for pT4a carcinomas. The 5-year overall, recurrence-free, and disease-specific survival rates for stages III and IVa were 59, 65, and 81% (11). Pantazis et al. (12) achieved with TLM in 24 patients with pT3 supraglottic carcinomas a 5-year disease-specific survival and larynx preservation rate of 92%. Vilaseca et al. (14) published the largest series of 128 patients with pT3 and 25 patients with pT4a supraglottic carcinomas treated with TLM. The 5-year laryngectomy-free survival was 75%. The 5-year overall survival rate was 56%.

In the recent literature, only a few publications can be found on oncologic results of open-neck SGL and supracricoid partial laryngectomy for supraglottic carcinomas. For open-neck SGL in T1–T3 supraglottic carcinomas, 5-year local control rates from 90 to 93% and overall survival rates from 52 to 75% have been reported (35–37). Approximately 10% patients need secondary laryngectomy because of aspiration (37). With extended open-neck SGL for T3 carcinomas, local control rates from 71 to 94% have been published (38, 39). Schwaab et al. (40) reported on a larger series of patients, mainly with T2 and T3 tumors, who had supracricoid partial resection with cricohyoidopexy. Only 4% patients experienced local recurrence. Post-operative aspiration was a relevant problem in 19% patients, and 9% needed laryngectomy for functional reasons. The 5-year overall survival rate was 88%. In summary, it could be shown that the oncologic results achieved by us are comparable to other TLM series, to open-neck SGL and to supracricoid partial resection.

In 2003, the RTOG 91-11 trial showed better locoregional control and larynx preservation for concurrent chemoradiotherapy than for induction chemotherapy followed by RT and by RT alone (16). Since that time, in many cancer centers, concurrent chemoradiotherapy has become the preferred non-surgical treatment for larynx preservation in locally advanced supraglottic carcinomas, despite its toxicity. Even though more than 80% of the patients in the RTOG 91-11 trial had a Karnofsky index of >90, only 70% of patients could complete the concurrent chemoradiotherapy protocol due to toxicity. A high number of patients (82%) suffered from severe toxicity, and 5% died of therapy-associated complications. Thus, therapy-related mortality is significantly higher when concurrent chemoradiotherapy is used for larynx preservation compared to the surgical approaches. Long-term results of the RTOG 91-11 trial were published in 2013 (17). It was remarkable that in the concurrent chemoradiotherapy arm of the trial, late deaths unrelated to larynx cancer occurred. The cause of death in these patients is not known and it was speculated that some might be due to late-toxicity such as swallowing dysfunction and (silent) aspiration (17).

### Complications

For the evaluation of a surgical procedure, the complication rate is an important factor. In our series, 2.2% patients experienced post-operative endolaryngeal hemorrhage, requiring coagulation in general anesthesia. The incidence of post-operative hemorrhage is reported between 4 and 13% in different TLM series (9, 10, 12–14). The lower incidence experienced by us is attributed to consequent liger clipping of both supralaryngeal arteries. Another complication occurring frequently after supraglottic resection is aspiration pneumonia. In our cohort, the incidence was 4%. In other TLM series, the incidence varies between 2 and 12% (9, 10, 13, 14, 41). The risk of aspiration after open-neck SGL and supracricoid partial resection is even higher. The reported aspiration risk ranges from 3.8% to 19% for open-neck surgery (35, 37–40). After supracricoid partial resection, the risk of severe aspiration followed by lung complications increases with increasing age and pre-existing lung disease. This explains why patients older than 60 years are often excluded from supracricoid partial resection with cricohyoidopexy (42).

In our patient cohort, a total of 12 (13.2%) patients had temporary tracheostomy; five patients were tracheotomized electively and seven patients due to various complications. The incidence of temporary tracheostomy in different TLM series varies between 4 and 45.8% (8–10, 12). This variation may reflect different patient populations and different indications for prophylactic tracheostomy. The prevalence of permanent tracheostomy is reported below 3% (9, 10, 12, 14).

### Functional Outcomes

A functioning larynx could be preserved in 89% (81/91) patients in our cohort. According to T category, larynx preservation was possible in 91% of patients with pT1, 97% of patients with pT2, 89% of patients with pT3, and 69% of patients with pT4a tumors. At the end of follow-up, no patient had a tracheostoma, two patients needed permanent PEG feeding (those patients declined laryngectomy), and one patient underwent total laryngectomy because of severe aspiration. All authors agree that swallowing rehabilitation is quicker and better following TLM than open-neck SGL (42). The reported rate of secondary laryngectomies in TLM series is 0–4%, and the rate of permanent PEG feeding is 0–2% (9, 10, 12, 14, 43). The frequency of laryngectomy for persistent aspiration after open-neck SGL ranges between 3.5 and 12.5% (42).

### Patient-Reported Outcomes

Quality of life questionnaires are routinely used in clinical trials. In contrast, it is uncommon to ascertain QoL data in clinical practice. Since our patient cohort was not treated in a prospective trial, baseline functional assessments preoperatively were not done. We performed a cross-sectional study and administered three QoL instruments to the disease-free long-term survivors of our cohort. The study was done median 88.5 months (range, 25–135 months) after completion of the cancer treatment. Normalcy of diet has the highest magnitude for patients, and our cohort achieved excellent outcomes with a median score of 91. However, three of the 36 (8.3%) patients perceived unsatisfactory outcomes with a PSS-HN score of 50. Restrictions in the domain “normalcy of diet” were due to discomfort caused by xerostomia. The dysphagia-related and voice-related QoL was examined with post-treatment MDADI and VHI scores. The median scores for our group of long-term survivors indicated that patients perceive low levels of swallowing and voice impairment. We could see, however, a clearly negative influence of post-operative RT on both swallowing and voice-related QoL. Today, the standard technique for adjuvant RT is IMRT and better preservation of the salivary glands and reduction of the radiation dose to uninvolved swallowing structures hopefully will translate in better swallowing- and voice-related QoL.

### Limitations

The present study has some limitations. First, this study is a retrospective outcomes analysis, although the data have been recorded prospectively. In addition, the study included a limited number of patients, particularly the cross-sectional study of swallowing- and voice-related QoL. The fact that QoL could only be examined in the highly selected subgroup of long-term survivors could be a potential selection bias. In order to address these limitations, additional studies with larger sample-size are needed to more definitely assess oncologic and functional outcomes of TLM vs. alternative treatment strategies.

### Conclusion

In conclusion, our results suggest that TLM can be considered an option for surgical larynx preservation in early and selected locally advanced supraglottic carcinomas. The oncologic results are comparable to open-neck SGL ± adjuvant (chemo-)radiotherapy and to definitive chemoradiotherapy. A functioning larynx can be preserved in high numbers of patients. Tracheostomy and gastrostomy can be avoided in most cases. Patient-reported outcomes demonstrate that long-term survivors perceive low levels of QoL impairment and functional deterioration over time does not occur.

## Ethics Statement

The study was approved by the Ethics Committee of the Medical Faculty of the Christian-Albrechts-University of Kiel, Number D 417/14.

## Author Contributions

PA contributed to the conception of the work, data analysis and interpretation, manuscript writing, and final approval of the version to be published. MG-D contributed to the data collection and analysis and drafting the manuscript. AF contributed to the data collection and analysis and drafting the manuscript. CS contributed to the data analysis. JH contributed to the statistical calculations.

## Conflict of Interest Statement

The authors declare that the research was conducted in the absence of any commercial or financial relationships that could be construed as a potential conflict of interest.

## References

[1] Steiner W. Results of curative laser microsurgery of laryngeal carcinomas. Am J Otolaryngol (1993) 14:116–21. 10.1016/0196-0709(93)90050-H 8484476

[2] Ambrosch P Kron M Steiner W. Carbon dioxide laser microsurgery for early supraglottic carcinoma. Ann Otol Rhinol Laryngol (1998) 107:680–8. 10.1177/000348949810700810 9716871

[3] Swanson MS Low G Sinha UK Kokot N. Transoral surgery vs intensity-modulated radiotherapy for early supraglottic cancer: a systematic review. Curr Opin Otolaryngol Head Neck Surg (2017) 25:133–41. 10.1097/MOO.0000000000000345 28106658

[4] Iro H Waldfahrer F Altendorf-Hofmann A Weidenbecher M Sauer R Steiner W Transoral laser surgery of supraglottic cancer. Arch Otolaryngol Head Neck Surg (1998) 124:1245–50. 10.1001/archotol.124.11.1245 9821928

[5] Grant DG Salassa JR Hinni ML Pearson BW Hayden RE Perry WC. Transoral laser microsurgery for carcinoma of the supraglottic larynx. Otolaryngol Head Neck Surg (2007) 136:900–6. 10.1016/j.otohns.2006.12.015 17547977

[6] Agrawal A Moon J Davis RK Sakr WA Giri SPG Valentino J Transoral carbon dioxide laser supraglottic laryngectomy and irradiation in stage I, II, and III squamous cell carcinoma of the supraglottic larynx. Arch Otolaryngol Head Neck Surg (2007) 133:1044–50. 10.1001/archotol.133.10.1044 17938330

[7] Bussu F Almadori G De Corso E Rizzo D Rigante M Parrilla C Endoscopic horizontal partial laryngectomy by CO2 laser in the management of supraglottic squamous cell carcinoma. Head Neck (2009) 31:1196–206. 10.1002/hed.21085 19360749

[8] Peretti G Piazza C Ansarin M De Benedetto L Cocco D Cattaneo A Transoral CO2 laser microsurgery for Tis-T3 supraglottic squamous cell carcinomas. Eur Arch Otorhinolaryngol (2010) 267:1735–42. 10.1007/s00405-010-1284-1 20499077

[9] González-Márquez R Rodrigo JP Llorente JL Alvarez-Marcos C Díaz JP Suárez C Transoral CO2 laser surgery for supraglottic cancer. Eur Arch Otorhinolaryngol (2012) 269:2081–6. 10.1007/s00405-012-2016-5 22484514

[10] Canis M Martin A Ihler F Wolff HA Kron M Matthias C Results of transoral laser microsurgery for supraglottic carcinoma in 277 patients. Eur Arch Otorhinolaryngol (2013) 270:2315–26. 10.1007/s00405-012-2327-6 23306348 PMC3699705

[11] Canis M Ihler F Martin A Wolff HA Matthias C Steiner W. Results of 226 patients with T3 laryngeal carcinoma after treatment with transoral laser microsurgery. Head Neck (2014) 36:652–9. 10.1002/hed.23338 23596018

[12] Pantazis D Liapi G Kostarelos D Kyriazis G Pantazis TL Riga M. Glottic and supraglottic pT3 squamous cell carcinoma: outcomes with transoral laser microsurgery. Eur Arch Otorhinolaryngol (2015) 272:1983–90. 10.1007/s00405-015-3611-z 25821031

[13] Vilaseca I Bernal-Sprekelsen M Blanch L. Transoral laser microsurgery for T3 laryngeal tumors: prognostic factors. Head Neck (2010) 32:929–38. 10.1002/hed.21288 19953612

[14] Vilaseca I Blanch JL Berenguer J Grau JJ Verger E Muxí Á Transoral laser microsurgery for locally advanced (T3-T4a) supraglottic squamous cell carcinoma: sixteen years of experience. Head Neck (2016) 38:1050–7. 10.1002/hed.24408 26872432

[15] Mannelli G Lazio MS Luparello P Gallo O. Conservative treatment for advanced T3-T4 laryngeal cancer: meta-analysis of key oncological outcomes. Eur Arch Otorhinolaryngol (2018) 275:27–38. 10.1007/s00405-017-4799-x 29119321

[16] Forastiere AA Goepfert H Maor M Pajak TF Weber R Morrison W Concurrent chemotherapy and radiotherapy for organ preservation in advanced laryngeal cancer. N Engl J Med (2003) 349:2091–8. 10.1056/NEJMoa031317 14645636

[17] Forastiere AA Zhang Q Weber RS Maor MH Goepfert H Pajak TF Long-term results of RTOG 91-11: a comparison of three nonsurgical treatment strategies to preserve the larynx in patients with locally advanced larynx cancer. J Clin Oncol (2013) 31:845–52. 10.1200/JCO.2012.43.6097 23182993 PMC3577950

[18] Forastiere AA Ismaila N Lewin JS Nathan CA Adelstein DJ Eisbruch A Use of larynx-preservation strategies in the treatment of laryngeal cancer: American Society of Clinical Oncology clinical practice guideline update. J Clin Oncol Nov (2017) 27:JCO2017757385 10.1200/JCO.2017.75.7385

[19] Machtay M Moughan J Trotti A Garden AS Weber RS Cooper JS Factors associated with severe late toxicity after concurrent chemoradiation for locally advanced head and neck cancer: an RTOG analysis. J Clin Oncol (2008) 26:3582–9. 10.1200/JCO.2007.14.8841 18559875 PMC4911537

[20] Hoffman HT Porter K Karnell JH Cooper JS Weber RS Langer CJ Laryngeal cancer in the United States: changes in demographics, patterns of care, and survival. Laryngoscope (2006) 116(Suppl 111):1–13. 10.1097/01.mlg.0000236095.97947.26 16946667

[21] Chen AY Halpern M. Factors predictive of survival in advanced laryngeal cancer. Arch Otolaryngol Head Neck Surg (2007) 133:1270–6. 10.1001/archotol.133.12.1270 18086971

[22] Garcia Lorenzo J Montoro Martinez V Rigo Quera A Codina Aroca A Lopez Vilas M Quer Agusti M Modifications in the treatment of advanced laryngeal cancer throughout the last 30 years. Eur Arch Otorhinolaryngol (2017) 274:3449–55. 10.1007/s00405-017-4639-z 28625009

[23] Steiner W Ambrosch P Endoscopic Laser Surgery of the Upper Aerodigestive Tract. Stuttgart, New York: Thieme (2000).

[24] Remacle M Hantzakos A Eckel H Evrard AS Bradley PJ Chevalier D Endoscopic supraglottic laryngectomy: a proposal for a classification by the working committee on nomenclature, European Laryngological Society. Eur Arch Otorhinolaryngol (2009) 266:993–8. 10.1007/s00405-008-0901-8 19130072

[25] Chen AY Frankowski R Bishop-Leone J Hebert T Leyk S Lewin J The development and validation of a dysphagia-specific quality-of-life questionnaire for patients with head and neck cancer. Arch Otolaryngol Head Neck Surg (2001) 127:870–6. 11448365

[26] Jacobson BH Johnson A Grywalski C Silbergleit A Jacobson G Benninger MS The voice handicap index (VHI): development and validation. Am J Speech Lang Pathol (1997) 6:66–70. 10.1044/1058-0360.0603.66

[27] List MA Ritter-Sterr C Lansky SB. A performance status scale for head and neck cancer patients. Cancer (1990) 66:564–9. 10.1002/1097-0142(19900801)66:3<564::AID-CNCR2820660326>3.0.CO;2-D 2364368

[28] List MA D’Antonio LL Cella DF Siston A Mumby P Haraf D The performance status scale for head and neck cancer patients and the functional assessment of cancer therapy-head and neck scale: a study of utility and validity. Cancer (1996) 77:2294–301. 10.1002/(SICI)1097-0142(19960601)77:11<2294::AID-CNCR17>3.0.CO;2-S 8635098

[29] Bernier J Domenge C Ozsahin M Matuszewska K Lefebvre JL Greiner RH Postoperative irradiation with or without concomitant chemotherapy for locally advanced head and neck cancer. N Engl J Med (2004) 350:1945–1052. 10.1056/NEJMoa032641 15128894

[30] Cooper JS Pajak TF Forastiere AA Jacobs J Campbell BH Saxman SB Postoperative concurrent radiotherapy and chemotherapy for high-risk squamous cell carcinoma of the head and neck. N Engl J Med (2004) 350:1937–44. 10.1056/NEJMoa032646 15128893

[31] Cooper JS Zhang Q Pajak TF Forastiere AA Jacobs J Saxman SB Long-term follow-up of the RTOG 9501/Intergroup phase III trial: postoperative concurrent radiation therapy and chemotherapy in high-risk squamous cell carcinoma of the head & neck. Int J Radiat Oncol Biol Phys (2012) 84:1198–205. 10.1016/j.ijrobp.2012.05.008 22749632 PMC3465463

[32] Roh JL Kim DH Park CI Voice, swallowing and quality of life in patients after transoral laser surgery for supraglottic carcinomas. J Surg Oncol (2008) 98:184–9. 10.1002/jso.21101 18561157

[33] Karatzanis AD Psychogios G Zenk J Waldfahrer F Hornung J Velegrakis GA Evaluation of available surgical management options for early supraglottic cancer. Head Neck (2010) 32:1048–55. 10.1002/hed.21289 19953613

[34] Ambrosch P Rödel R Kron M Steiner W Die transorale Lasermikrochirurgie des Larynxkarzinoms. Eine retrospektive Analyse von 657 Patientenverläufen. Onkologe (2001) 7:505–12. 10.1007/s007610170103

[35] Prades JM Simon PG Timoshenko AP Dumollard JM Schmitt T Martin C. Extended and standard supraglottic laryngectomies: a review of 110 patients. Eur Arch Otorhinolaryngol (2005) 262:947–52. 10.1007/s00405-004-0882-1 16362268

[36] Bron LP Soldati D Monod ML Megevand C Brossard E Monnier P Horizontal partial laryngectomy for supraglottic squamous cell carcinoma. Eur Arch Otorhinolaryngol (2005) 262:302–6. 10.1007/s00405-004-0824-y 15316823

[37] Sevilla MA Rodrigo JP Llorente JL Cabanillas R Lopez F Suarez C. Supraglottic laryngectomy: analysis of 267 cases. Eur Arch Otorhinolaryngol (2008) 265:11–6. 10.1007/s00405-007-0415-9 17684753

[38] Scola B Fernandez-Vega M Martinez T Fernandez-Vega S Ramirez C Management of cancer of the supraglottis. Otorhinolaryngol Head Neck Surg (2001) 124:195–8. 10.1067/mhn.2001.112202 11226956

[39] Herranz J Martinez-Vidal J Martinez Moran A Supraglottic laryngectomy. Still on-going. Acta Otorrinolaryngol Esp (2006) 57:235–41. 10.1016/S0001-6519(06)78699-X 16768202

[40] Schwaab G Kolb F Julieron M Janot F Le Ridant AM Mamelle G Subtotal laryngectomy with cricohyoidopexy as first treatment procedure for supraglottic carcinoma: Institut Gustave-Roussy experience (146 cases, 1974-1997). Eur Arch Otorhinolaryngol (2001) 258:246–9. 10.1007/s004050100348 11548904

[41] Piazza C Barbieri D Del Bon F Grazioli P Perotti P Paderno A Functional outcomes after different types of transoral supraglottic laryngectomy. Laryngoscope (2015) 126:1131–5. 10.1002/lary.25562 26577372

[42] Ambrosch P Fazel A. Functional organ preservation in laryngeal and hypopharyngeal cancer. GMS Curr Top Otorhinolaryngol Head Neck Surg (2011) 10:Doc02. 10.3205/cto000075 22558052 PMC3341579

[43] Butler A Rigby MH Scott J Trites J Hart R Taylor SM. A retrospective review in the management of T3 laryngeal squamous cell carcinoma: an expanding indication for transoral laser microsurgery. J Otolaryngol Head Neck Surg (2016) 45:34–9. 10.1186/s40463-016-0147-1 27233357 PMC4884416

